# Idiopathic Spontaneous Hemorrhage of the Right Colic Artery: A Rare Vascular Emergency

**DOI:** 10.7759/cureus.92880

**Published:** 2025-09-21

**Authors:** Guy Loic Nguefang Tchoukeu, Ijeoma Nwankwo, Tracy Chukwu, Edgar M Luna Landa, Gregory Bartha

**Affiliations:** 1 Internal Medicine, Texas Tech University Health Sciences Center, Odessa, USA

**Keywords:** anticoagulation, endovascular embolization, hemoperitoneum, idiopathic spontaneous intraperitoneal hemorrhage, right colic artery rupture

## Abstract

Spontaneous rupture of the right colic artery is an exceptionally rare cause of acute intra-abdominal bleeding, especially in the absence of trauma. Prompt recognition and intervention are critical, particularly in anticoagulated patients, to prevent hemodynamic collapse and death. We present the case of a 64-year-old man with a history of atrial fibrillation on apixaban who presented with sudden-onset severe abdominal pain, hypotension, and tachycardia. A CT of the abdomen demonstrated intraperitoneal hematoma with active arterial extravasation from a distal branch of the right colic artery arising from the superior mesenteric artery (SMA). The patient was administered prothrombin complex concentrate and IV fluids and underwent emergent superselective interventional radiology (IR)-guided embolization of the bleeding vessel. Spontaneous right colic artery rupture can be life-threatening, especially in anticoagulated patients. Early CT scans and IR intervention can be life-saving and avoid the need for exploratory surgery. Clinicians should maintain a high index of suspicion for visceral artery hemorrhage in patients presenting with acute abdomen.

## Introduction

Spontaneous rupture of a visceral artery in the absence of trauma is a rare and potentially life-threatening cause of hemoperitoneum requiring urgent intervention [1]. Among visceral arteries, the splenic, hepatic, and superior mesenteric arteries (SMAs) are more frequently involved and usually occur in a context of aneurysm or pseudoaneurysm [2]. The rupture of the colic artery is extremely rare and sparsely documented in the literature.

The right colic artery is a branch of the SMA that supplies the ascending colon and right colonic flexure [3]. Rupture of this artery may result in retroperitoneal hematoma and colon ischemia that presents as abdominal pain and rapid hemodynamic deterioration. Timely identification of the bleeding source through imaging and urgent intervention are critical. Endovascular embolization has emerged as a safe and effective alternative to surgery in achieving hemostasis in such cases [4].

We describe a rare presentation of spontaneous rupture of the right colic artery in a patient with multiple chronic conditions on long-term anticoagulation. We will discuss diagnostic approaches and the role of endovascular intervention with superselective embolization for the management. Given the rarity and nonspecific presentation of this vascular emergency, this case underscores the need for clinicians to consider atypical intra-abdominal sources of hemorrhage in anticoagulated patients and the critical role of imaging and timely interventional radiology (IR) in the management.

## Case presentation

A 64-year-old man with a complex medical history, including hypertension, chronic atrial fibrillation (CHA₂DS₂-VASc score of 4 and HAS-BLED score of 2) on chronic anticoagulation with apixaban 5 mg twice daily, peripheral arterial disease, chronic obstructive pulmonary disease (COPD), rheumatoid arthritis, and heart failure with reduced ejection fraction (EF; 25-30%), presented to the emergency department (ED) with the acute onset of abdominal pain.

The patient reported a one-day history of severe abdominal pain that had begun suddenly the evening prior to admission, with no identifiable inciting event. He described the abdominal pain as gradually worsening and persistent. He also reported a possible syncopal episode and dizziness on the morning of admission. He denied associated symptoms such as nausea, vomiting, diarrhea, chest pain, dyspnea, fever, and chills. His last dose of apixaban was taken on the morning of presentation to the ED.

On initial evaluation, the patient was hypotensive (blood pressure 84/55 mmHg) and tachycardic (heart rate 125 bpm). His temperature was 37.5°C, and oxygen saturation was 98% on room air, with abdominal pain rated 8/10 in severity. Physical examination was remarkable for generalized abdominal tenderness without guarding. Laboratory studies (Table 1) were significant for leukocytosis (white blood cell count (WBC) 15.3 × 10⁹/L), anemia (hemoglobin 8.2 g/dL), and normal platelet count (239 × 10³/µL). Renal function, thyroid panel, liver enzymes, and electrolytes were within normal limits. Coagulation studies showed an international normalized ratio (INR) of 1.11, prothrombin time (PT) of 14.3 seconds, and partial thromboplastin time (PTT) of 24.8 seconds. An electrocardiogram (ECG) showed atrial fibrillation with rapid ventricular response (RVR; heart rate 128 bpm).

**Table 1:** Trend of selected laboratory parameters from admission to discharge This table summarizes the laboratory values of the patient from day 1 to day 5 of discharge. Parameters include complete blood count, coagulation profile, renal function, and electrolytes. WBC: White blood cell count; INR: International normalized ratio; PT: Prothrombin time; PTT: Partial thromboplastin time; BUN: Blood urea nitrogen

Laboratory	Day 1	Day 3	Day 5	Reference range
Hemoglobin (g/dL)	8.2	8	8.1	Men: 13.5–17.5; Women: 12.0–15.5
Hematocrit %	29	25	30	Men: 38.3%–48.6%; Women: 35.5%–44.9%
WBC (x10^3/mcL)	15.3	15.7	17.3	4.0–11.0 × 10³/μL
Platelets (x10^3/mcL)	239	251	245	150–450 × 10³/μL
INR	1.1	-	1.1	0.8–1.2 (not on anticoagulation)
PT (sec)	14.3	-	13	11–13.5 seconds
PTT (sec)	24.8	-	25	25–35 seconds
BUN (mg/dL)	30	22	17	7–20 mg/dL
Creatinine (mg/dL)	1.3	1.2	1.2	Men: 0.74–1.35; Women: 0.59–1.04
Sodium (mmol/L)	141	142	142	135–145 mmol/L
Potassium (mmol/L)	3.9	3.9	4.3	3.5–5.0 mmol/L

**Figure 1:**
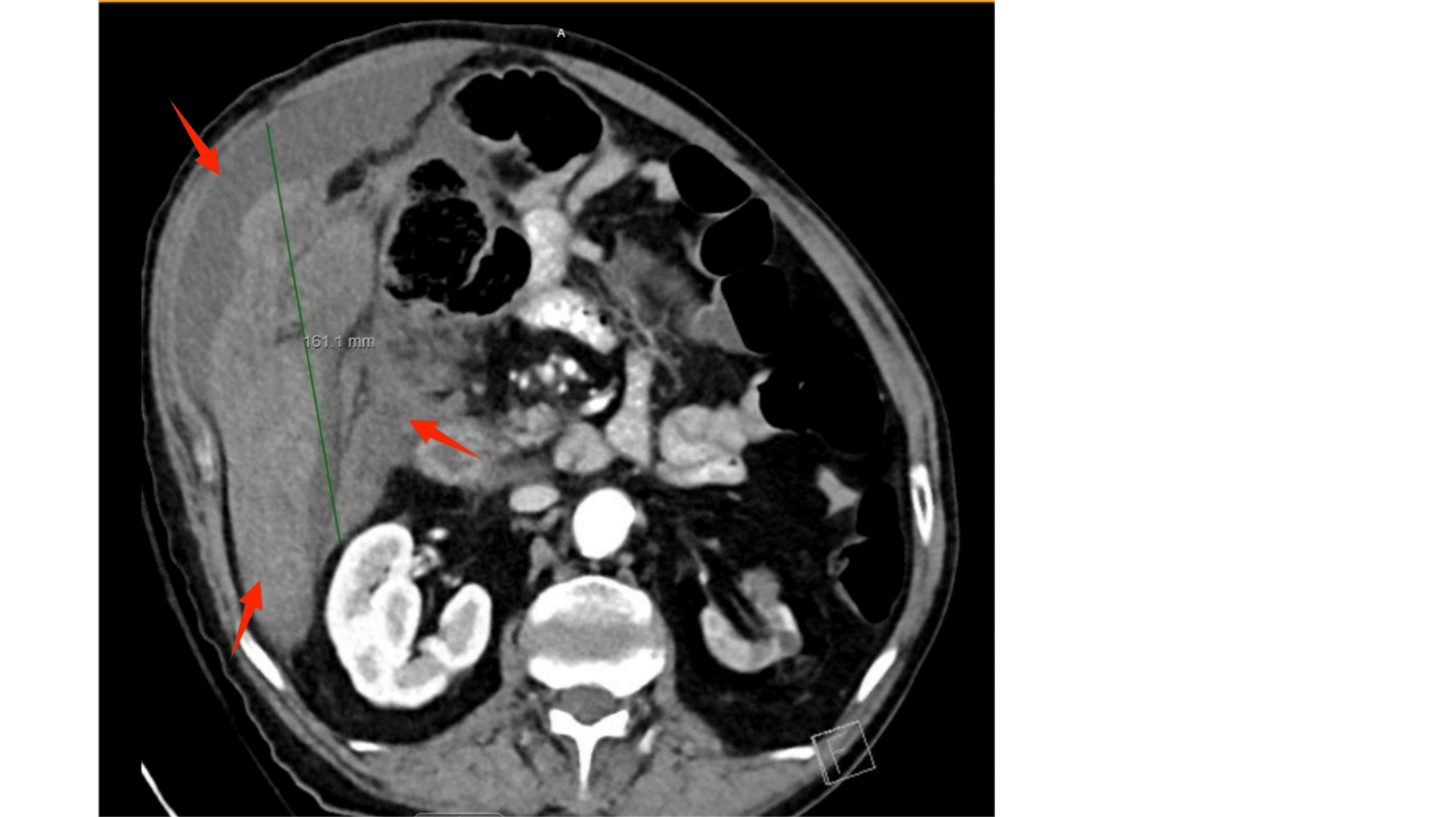
Axial abdominal CT scan showing large right intraperitoneal hematoma Contrast-enhanced axial CT image demonstrates a large right-sided intraperitoneal hematoma measuring approximately 161 mm (16.1 cm) in craniocaudal length. The arrows indicate hematoma.

Immediate resuscitation was initiated with two liters of crystalloids, resulting in stabilization of blood pressure. A contrast-enhanced CT scan of the abdomen and pelvis revealed a large 16-cm hematoma located posterior to and surrounding the mid-right ascending colon, extending along the right abdomen, surrounding the liver, and down to the pelvis along the paracolic gutter. Small foci of active hemorrhage were identified, likely originating from a branch of the right colic artery arising from the SMA. Additional findings included chronic atrophic changes in the left kidney (Figure 1).

**Figure 2:**
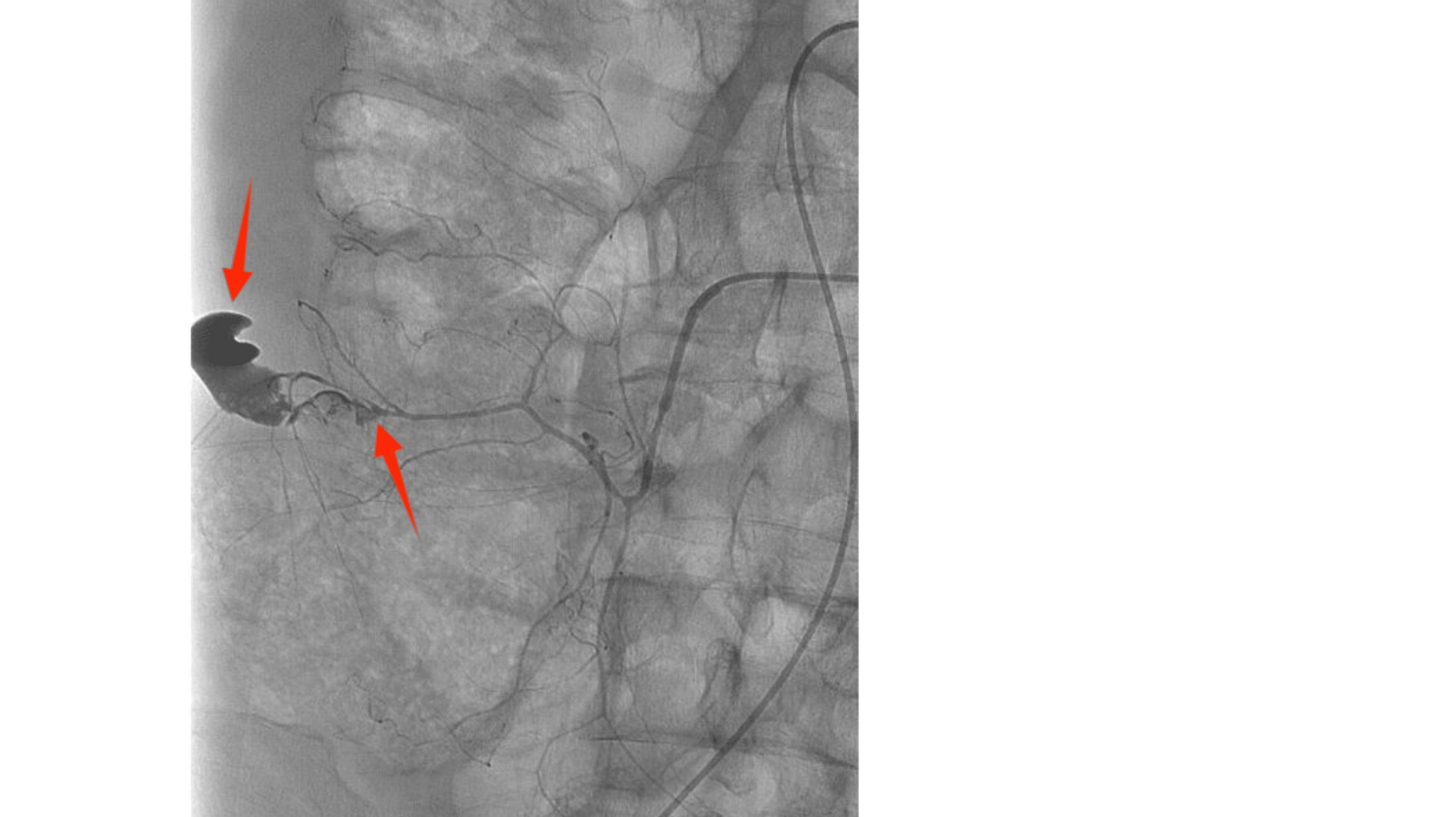
Angiography demonstrating active extravasation from a right colic artery branch Image of mesenteric angiography revealing active contrast extravasation from a distal branch of the right colic artery, confirming the source of ongoing hemorrhage. The arrows indicate the source of bleeding from the vessel.

The patient was urgently administered 2,000 units of prothrombin complex concentrate due to the recent dose of apixaban to reverse anticoagulation and prevent further bleeding. IR and the surgical team were consulted. The interventional radiologist recommended a CT mesenteric angiogram, given the patient’s hemodynamic stability. Initial angiographic images demonstrated active contrast extravasation from a branch of the right colic artery (Figure 2).

**Figure 3:**
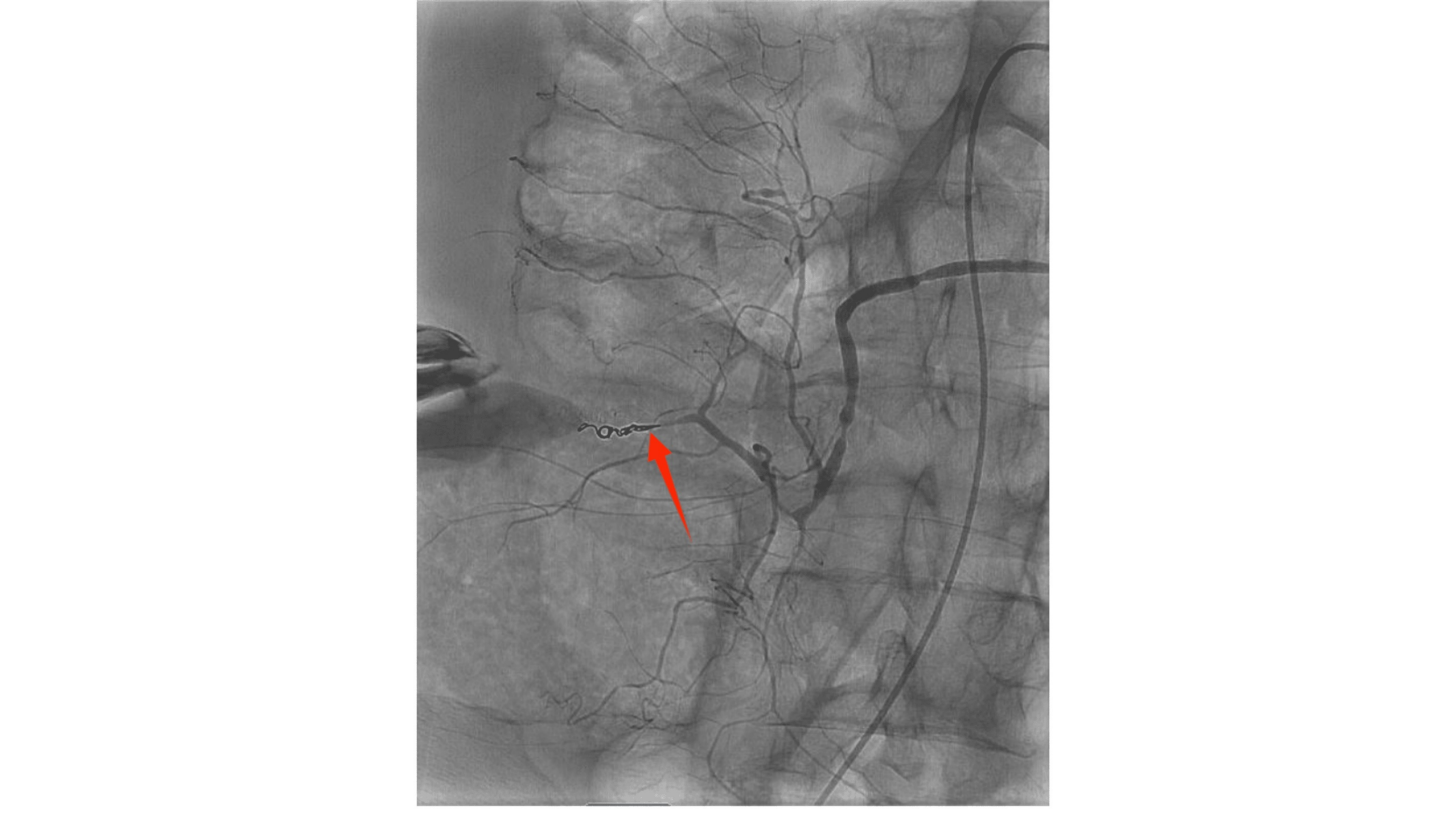
Post-embolization angiography showing successful hemostasis of right colic artery branch Follow-up mesenteric angiogram after embolization reveals complete cessation of contrast extravasation from the previously bleeding branch of the right colic artery. The arrow on the figure indicates the coil inserted to stop hemorrhage.

A 5 French glide catheter was advanced into the right colic artery branch and further into a fourth-order branch. At this point, the catheter could not be advanced further, so a 0.021-inch microcatheter was navigated to the site of hemorrhage. The bleeding artery was embolized to stasis using two 2-mm Interlock detachable coils, resulting in complete cessation of flow and no residual extravasation (Figure 3). No vascular abnormalities, such as aneurysms, were identified, and there were no immediate complications. Hemostasis at the right groin access site was achieved using an ExoSeal vascular closure device. The hematoma was left in place.

Following the procedure, the patient was admitted to the ICU for close monitoring. He was started on an amiodarone infusion due to atrial fibrillation with RVR, and anticoagulation was held. The patient converted to normal sinus rhythm by day 2, and his abdominal pain significantly improved by day 3. He was then transferred to the general medical ward and was discharged on day 5 with a recommendation to follow up with surgical and primary care. 

## Discussion

Idiopathic spontaneous intraperitoneal hemorrhage (ISIH), or abdominal apoplexy, refers to hemorrhage from visceral vessels in the absence of any clearly defined pathology [5]. The present case illustrates a unique instance of spontaneous rupture of a distal branch of the right colic artery, an extremely rare source of atraumatic, non-aneurysmal bleeding. To our knowledge, such an event, particularly in the setting of chronic anticoagulation, has been rarely documented in the literature.

The diagnosis can be complex, as ISIH often mimics other intra-abdominal pathologies, with presentations ranging from vague abdominal discomfort to profound hemorrhagic shock. In our patient, the clinical course closely followed Osunkunle’s three-phase model of hemoperitoneum: an initial phase of severe pain, a latent asymptomatic interval, and a terminal phase marked by worsening pain and hypotension [4].

ISIH typically affects men more frequently than women (3:1 ratio), with peak incidence between 40 and 70 years of age [6,7]. Risk factors include hypertension and atherosclerosis, both of which were present in our patient [1,6]. These conditions may weaken the vascular media and predispose vessels to rupture under transient spikes in pressure. Although no aneurysm was identified on imaging, histological studies suggest that such spontaneous ruptures can occur in structurally abnormal vessels that appear radiographically normal [8]. Chronic inflammatory diseases, such as rheumatoid arthritis, may further contribute to microvascular fragility through ongoing endothelial damage or vasculitis mechanisms, though this is rarely confirmed in the absence of specimens. While apixaban is associated with a lower risk of major bleeding compared to warfarin, it does not eliminate the possibility of spontaneous hemorrhage, particularly in elderly patients with multiple comorbidities and fragile vasculature. In this case, no underlying vascular malformation was found, supporting the idiopathic nature of the event. This is consistent with prior reports describing ruptures in histologically weakened but radiographically normal vessels [8].

CT angiography remains the diagnostic modality of choice, enabling prompt visualization of active bleeding and guiding therapeutic intervention. In our case, superselective embolization was successfully performed, allowing for hemorrhage control without surgical exploration. This is increasingly becoming the preferred management strategy in hemodynamically stable or stabilized patients, as it reduces morbidity associated with laparotomy and permits targeted therapy [8]. Focused Assessment with Sonography in Trauma (FAST) scan may serve as an alternative to CT scan in unstable patients to rapidly detect intra-abdominal fluid in the ED, though it lacks the detailed anatomical information needed to localize the bleeding vessel [7]. Pre-procedural reversal of anticoagulation with Kcentra (prothrombin complex concentrate) was essential for hemostasis, and ICU monitoring allowed for optimal control of the patient’s atrial fibrillation and gradual recovery.

ISIH should be considered in the differential diagnosis of elderly patients with cardiovascular comorbidities presenting with unexplained abdominal pain and hemodynamic instability in the absence of trauma. The use of anticoagulants, such as apixaban, may unmask underlying vascular fragility, predisposing patients to spontaneous hemorrhage. Effective management requires a multidisciplinary approach and timely coordination between emergency medicine, IR, and surgical teams to facilitate prompt diagnosis and targeted intervention.

## Conclusions

This case illustrates a spontaneous rupture of the right colic artery in an anticoagulated patient, presenting as acute abdominal pain with hemodynamic instability. CT imaging revealed a large pericolic hematoma with active arterial extravasation, and definitive management was achieved through superselective embolization without surgical intervention. The absence of trauma and the rarity of ISIH from the colic artery underscore the diagnostic challenge. This case reinforces the need for clinicians to consider visceral artery rupture in the differential diagnosis of acute abdomen, particularly in anticoagulated patients. Early recognition and prompt IR-guided intervention can be lifesaving and may prevent laparotomy.
